# A neonatal case of 3-hydroxy-3-methylglutaric-coenzyme A lyase deficiency

**DOI:** 10.1186/1824-7288-39-33

**Published:** 2013-05-24

**Authors:** Francesca Santarelli, Michela Cassanello, Ausilia Enea, Francesca Poma, Valentina D'Onofrio, Giovanna Guala, Giangiacomo Garrone, Paola Puccinelli, Ubaldo Caruso, Francesco Porta, Marco Spada

**Affiliations:** 1 Department of Pediatrics, University of Torino, Regina Margherita Children Hospital, Piazza Polonia 92, 10126 Torino, Italy; 2 Gaslini Hospital, Via Gerolamo Gaslini, 5, 16148Genova, Italy; 3 Neonatal Intensive Care Unite, Maria Vittoria Hospital, Via Cibrario 72, 10144 Torino, Italy; 4 Ivrea General Hospital, P.zza Credenza 2, 10015 Ivrea (Torino), Italy

## Abstract

3-hydroxy-3-methylglutaric aciduria (OMIM 246450) is a rare autosomal recessive inborn of metabolism due to the deficiency of 3-hydroxy-3-methylglutaryl-coenzyme A (HMG-CoA) lyase, an enzyme involved both in the ketogenic pathway and leucine catabolism. Acute decompensations present with lethargy, cianosis, hypotonia, vomiting and metabolic acidosis with hypoketotic hypoglycemia. We report the case of a 3 days male with sudden hypoglycemic crisis initially misdiagnosed as a sepsis. HMG-CoA lyase deficiency was achieved through acyl-carnitines profile (showing a typical increasing of 3-hydroxy-isovaleryl and 3-methylgluraryl carnitines) and urinary organic acids analysis (disclosing elevation of 3-hydroxy-3-methylglutaric, 3-methyl-glutaconic, 3-methylglutaric and 3-hydroxyisovaleric acids). This case underlines the need of suspecting such inborn metabolic disorder in cases with hypoglycemia and metabolic acidosis. Acyl-carnitine and urinary organic acids profiles are essential to achieve a prompt diagnosis of treatable metabolic disorders in order to prevent their acute crisis with serious or even fatal consequences.

## Introduction

3-hydroxy-3-methylglutaric aciduria (OMIM 246450) is a rare autosomal recessive inborn disease due to the deficiency of 3-hydroxy-3-methylglutaryl-coenzyme A (HMG-CoA) lyase, an enzyme catalyzing the cleavage of HMG-CoA to acetyl-CoA and acetoacetic acid, leading to the common final step of ketogenic pathway and leucine catabolism, presenting with a typical pattern of urinary organic acids. The disease was first described by Faull in 1976 and has an estimated prevalence of less than 1 in 100,000 live births [1,2].

It usually presents in the first year of life during a state of catabolism triggered by a prolonged fasting or intercurrent illness. The acute presentation includes vomiting, hypotonia, lethargy, metabolic acidosis, and non-ketotic hypoglycemia. Moreover, Reye-like syndrome with acute liver failure can occur [3,4]. Huge amounts of 3-hydroxy-3-methylglutaric, 3-methylglutaconic, 3-methylglutaric and 3-hydroxyisovaleiric acids characterize the biochemical pattern of the disease [3,5].

Here we report the case of a newborn with HMG-CoA lyase deficiency presenting at 3 days of life with a sudden severe hypoglycemic crisis misdiagnosed with sepsis.

## Case report

We describe the case of a male newborn, born at term from non-consanguineous parents by vaginal delivery, with an APGAR scores 10 and 10 at 5 and 10 minutes, respectively, and antropometric parameters at the 50° percentile. Since the mother’s bacteriological vaginal smear was positive for Streptococcum Beta Agalactiae, the mother underwent a complete intrapartum antibiotic prophylaxis (ampicillin and sulbactam 3 g five hours before delivery, then 1.5 g one hour before delivery). The discharge was carried out on the third day after delivery. Four hours after being discharged, the infant was admitted to a primary care hospital for poor suckling and sudden multiple episodes of hypotonia, oculogyration associated with hypothermia, progressive tachypnoea and respiratory failure.

Initial laboratory investigations revealed white blood cell count 14,140/μl, c-reactive protein of 0.33 mg/dl and severe hypoglycemia (3 mg/dl, reference range 70–110). Arterial blood gas analysis revealed a partially corrected metabolic acidosis (blood pH 7.226, pCO2 41.7 mmHg, pO2 47.2 mmHg, bicarbonate 16.9 mEq/l, base excess −10.3 mEq/l). Sepsis was initially suspected and the infant was put on endovenous hydratation (balanced pediatric solution for endovenous infusion – glucose content 55 g/L - 80 ml/kg/day, glucose infusion 3 mg/kg/min) and treated with antibiotic therapy (Ampicillin 25 mg/kg b.w. every 6 hours and Gentamicin 4 mg/Kg/day for 4 days).

During next four hours, the patient developed a progressive dyspnoea with worsening of the metabolic acidosis, so he was transferred to our department, where metabolic workup was carried out, revealing mild hyperammonaemia (63 μmol/L; reference range 11–32), hyperlactataemia (3.14 mmol/L; reference range 0.22-2.22) and moderate hypertransaminasemia (aspartate aminotransferases 52 U/l, alanine aminotransferases 39 U/L; reference range10-37). No ketone bodies were detected in urines.

Glycaemic levels were raised up by endovenous infusion but not normalized, measuring 30 mg/dl. Hormonal panel including cortisol (352.7 nmol/L, reference range 220.7-689.7), adrenocorticotropic hormone (32 ng/ml, reference range 7.9-66.1 ng/ml ), growth hormone (12 ng/ml, reference range 1–20 ng/ml), glucagon (102 ng/L, reference range 50–150 pg/ml), insulin (83.3 pmol/L, reference range 41.6-159.7) was normal. Imaging (cerebral, heart and abdominal ultrasound scan) did not reveal any evidence of heart, renal or cerebral malformations.

The patient was treated with endovenous 10% glucose administration (80 ml/kg/day, glucose amount 6.1 mg/kg/min) and bicarbonate (NaHCO3 total amount 1.6 mEq) until the recovery from hypoglycaemia and acidosis.

**Figure 1:**
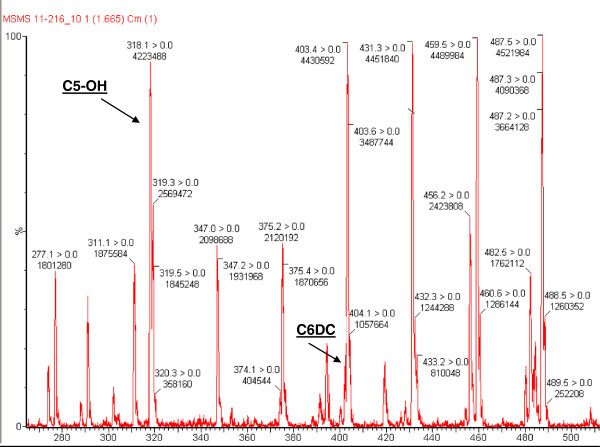
Acyl-carnitine profile revealing a typical increasing in 3-Hydroxy isovaleryl- carnitine (C5-OH) and 3-Methylglutaryl-carnitine (C6-DC) concentrations.

The evidence of severe hypoglycaemia accompanied by acidosis and hyperammonaemia prompted the differential diagnosis between beta-oxidation disorders and gluconeogenesis defects. A second level metabolic screening was then performed, including plasma aminoacids, bloodspot acyl-carnitine and urine organic acid analysis. Bloodspot acyl-carnitine assay showed significant increase of C5-OH acyl-carnitine (corresponding to the isobar compounds 2-hydroxy/3-hydroxy-isovaleryl-, 3-methylcrotonyl-, 2methyl-3hydroxy-butyryl- or 3-methylglutaconyl- carnitine) and of C6-DC acylcarnitine (3-methylglutaryl- or adipoyl – carnitine) (Figure 1). The simultaneous increase of these two markers led to identify them as 3-hydroxy-isovaleryl carnitine (7.78 μmol/l, reference range 0.00-0.40) and 3-methylglutaryl carnitine (0.76 μmol/l, reference range 0.00-0.14), respectively.

**Figure 2:**
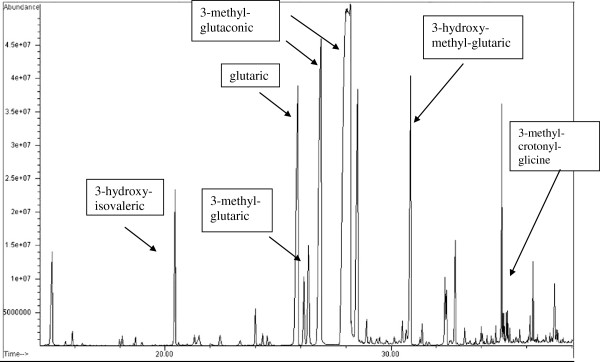
Urinary organic acids profile with evident increasing in 3-hydroxy-isovaleric, 3-methylglutaric, glutaric, 3-methyl-glutaconic, 3-hydroxy-3-methyl-glutaric acids and 3-methyl-crotonyl-glicine.

Urine organic acid analysis showed high excretion of 3-hydroxy-3-methyl-glutaric acid and 3-methyl-crotonyl-glycine (Figure 2; Table 1) confirming the bloodspot acylcarnitine abnormalities. This pattern led to the final diagnosis of HMG-CoA lyase deficiency.

**Table 1:** Characteristic urinary organic acids profile with evident increasing in 3-hydroxy-isovaleric, 3-methylglutaric, glutaric, 3-methyl-glutaconic, 3-hydroxy-3-methyl-glutaric acids and 3-methyl-crotonyl-glicine

**Organic acid**	**Concentration (μMol/mMol Creatinine)**	**Upper normal range (μMol/mMol Creatinine)**
3-hydroxy-3-methyl-glutaric	1028.4	50
3-methyl-crotonyl-glycine	27.6	2
3-hydroxy-isovaleric	1154.8	46
3-methyl-glutaric	348	7
glutaric	1274.9	5
3-methyl-glutaconic	4166.4	6

Therefore, infusion and antibiotic treatment were stopped and maintenance therapy was based on a low fat and protein diet with frequent meals in order to avoid hypoglycemia. Moreover, a carnitine supplement (400 mg/day) was started.

Four months after diagnosis, the patient presented another severe hypoglycemic episode after a viral infection which was solved out within 36 hours through hydration and glucose infusion. In spite of the severe episode of hypoglycaemia, no neuropsychomotor impairment is present to date. He is now twenty-two months old with normal development and growth.

## Discussion

3-hydroxy-3-methylglutaric aciduria is a rare disease characterized by the lack of 3-hydroxy-3-methylglutaryl-coenzyme A (HMG CoA) lyase (HL), an enzyme presenting into two different isoforms: the first one, located in mithocondria, catalyzes the conversion of HMG-CoA into acetoacetate and acetyl-CoA, while the second one, expressed in peroxisomes, is hypothesized to be involved in cholesterol synthesis and long-chain fatty acid degradation [2].

HL deficiency leads to the a reduced ability to synthesize ketones, which are a primary energy source for brain when glucose is not available and which are employed, in newborn period and suckling, as substrates for lipid synthesis in myelin [2].

HMG CoA Lyase deficiency usually presents with acute metabolic crisis in the early neonatal period, being 60-70% of cases reported during the first year of life and thirty percent of them occuring between the second and the fifth day of life [1,2]. Acute crisis, usually triggered by catabolism induced by fasting , illness, stress or excessive physical exercise, include hypothermia, vomiting, diarrhea, dehydration, hypotonia, cianosis, apnea and metabolic acidosis with hypoketotic hypoglycemia potentially progressing to coma. Patients can also develop a Reye-like syndrome with hyperammonemia, hepatomegaly and encelopathy [3,4]. Late-onset forms have been described, characterized by the possible development of hepatic steatosis, pancreatitis, white matter alterations (due both the damage resulting from recurrent hypoglycemic episodes and by the lack of myelin synthesis during suckling period) and dilated cardiomyopathy. This latter was hypotesized to result both from the lack of ketones as energy source, both from impaired ketogenesis, intracellular fatty acid accumulation and secondary carnitine deficiency, due to its esterification to 3-methylglutarylcarnitine. L-carnitine supplementation, by the increasing the urinary excretion of toxic metabolites, is considered essential in order to prevent the development of cardiomyopathy. It’s also thought to manage the transport of fatty acids to the mithocondria for oxidation and to be a free radical scavenger and a cofactor in the oxidation of long-chain fatty acids [2,4-6].

After the first year of life patients usually show a prolonged resistance to fasting and most of adults are symptoms free.

Our patient presented with hypoglycemia and acidosis leading to coma. First assessments posed the wrong hypothesis of sepsis, but the persistence of metabolic acidosis, together with further investigations excluding any renal, heart or cerebral pathology and revealing hyperammonaemia, hyperlactataemia and hypoketonemia, led to suspect an inborn metabolic disease. As it should always be done in the differential diagnosis of hypoglycemic crisis, congenital endocrine deficiencies were ruled out by finding normal concentrations of the hormones involved in glucose homeostasis (cortisol, adrenocorticotropic hormone, growth hormone, glucagon, insulin) [7].

Hypoketotic hypoglycemia is usually present in fatty acid oxidation defects, but it’s also associated with hyperinsulinism, endocrine deficiencies and ketogenesis defects. Determination of free fatty acids levels should be useful in order to differentiate these conditions. In reverse, ketoacidosis is a typical finding in organic acidurias (such as maple syrup urine disease, propionic aciduria, methylmalonic aciduria, isovaleric aciduria), gluconeogenesis defects (such as fructose-1, 6-bisphosphatase, pyruvatecarboxylase, phosphoenolpyruvate carboxykinase deficiencies), glycogenolysis, ketolysis and respiratory chain defects (Table 2).

**Table 2:** Differential diagnosis of hypoglycaemia

**Hypoketotic hypoglycaemia**		**Ketotic hypoglycaemia**
**Low free fatty acids**	**High free fatty acids**	
Hyperinsulinism	FAO defects	Sepsis
		Organic acidurias
Endocrine deficiencies: ACTH, cortisol, GH, glucagon	Ketogenesis defects	Gluconeogenesis defects
		Glycogenolysis defects
		Ketolysis defects
		Respiratory chain defects

However, it’s essential to perform acyl-carnitines and urinary organic acids profile in order to achieve a final diagnosis and to differentiate among fatty acid oxidation and ketogenesis defects (Glutaric aciduria type II, carnitine palmitoyltransferase II, carnitine acylcarnitine translocase, deficiency of very-long-chain acyl CoA dehydrogenase , medium-chain acyl-CoA dehydrogenase, 3-OH long-chain acyl CoA dehydrogenase, HMG-CoA lyase and HMG-CoA synthase).

## Conclusion

This case stresses the need for prompt recognition of this rare metabolic disorder in any case of newborn hypoglycemia with hypoketonemia. Acyl-carnitine and urinary organic acids profile are essential in order to achieve a quick diagnosis and to differentiate HL deficiency from organic acidemias and other fatty acid oxidation defects.

Furthermore, implementation of newborn screening procedures by tandem mass-spectrometry probably would have not avoided the acute crisis in our case, as the newborn screening results would have been available only after the discharge of the child. Routine newborn discharge within two or three days after delivery exposes apparently healthy children to the risk of development metabolic acute crisis despite newborn screening procedures.

Finally, it’s essential to suspect a metabolic inborn disease in every case of hypoglycaemia associated with metabolic acidosis. Acyl-carnitine and urinary organic acids profiles are essential to achieve a prompt diagnosis of all dietary treatable metabolic disorders in order to prevent their acute crisis with serious or even fatal consequences.

## Abbreviations

HMG-CoA: 3-hydroxy-3-methylglutaryl-coenzyme A; HL: 3-hydroxy-3-methylglutaryl-coenzyme A (HMG CoA) lyase.

## Competing interests

The authors declare that they have no competing interests.

## Authors’ contributions

MC, UC, PP, MS have given a substantial contribution to analysis and interpretation of data, and have been involved in drafting the manuscript. AE, VD, G. Garrone, G. Guala, F. Poma, F. Porta, MS have given a substantial contribution to : conception and design, acquisition and interpretation of data, and have been involved in the critical revision of the manuscript. All authors read and approved the final manuscript.
